# Comparison of the efficacy of five standard topical hemostats: a study in porcine liver and spleen models of surgical bleeding

**DOI:** 10.1186/s12893-020-00874-w

**Published:** 2020-09-25

**Authors:** Valerio Antonio Paternò, Alessandro Bisin, Alessandro Addis

**Affiliations:** 1 Mascia Brunelli S.p.A, Viale Monza 272, 20128 Milan, Italy; 2 CRABCC Animal Lab, Cremona, Italy

**Keywords:** Emosist, Cutanplast, Hemostat, Liver, Spleen, Surgical bleeding models

## Abstract

**Background:**

Several topical hemostats are available to help control surgical bleeding. Cutanplast is a highly absorbent and porous gelatin product that is available in Fast sponge and powder forms. This study investigated the hemostatic efficacy of Cutanplast Standard and Fast gelatin sponge and powder and Emosist oxidized regenerated cellulose (ORC) gauze in porcine liver and spleen surgical bleeding models.

**Methods:**

Cutanplast Standard and Fast gelatin sponge and Emosist ORC gauze were tested in liver abrasion/incision, liver puncture and spleen incision/puncture injuries, and Cutanplast Standard and Fast gelatin powder products were tested in liver abrasion/incision injuries. There were 13 liver injury (five abrasion, five incision and three puncture) and six spleen injury (three puncture and three incision sites) sites per animal.

**Results:**

Rapid hemostasis (≤ 2–5 min) was achieved in the liver abrasion and incision models with all Cutanplast gelatin sponge and powder products and Emosist ORC gauze, except in the liver incision model, time to hemostasis was > 5 min with Cutanplast Standard gelatin powder and Emosist ORC gauze. Rapid hemostasis occurred with Cutanplast Fast gelatin sponge and Emosist ORC gauze in the liver puncture and spleen puncture and incision models. In the spleen incision model, Cutanplast Standard gelatin sponge had a time to hemostasis approaching 10 min.

**Conclusion:**

Cutanplast gelatin sponge and powder products and Emosist ORC gauze may be suitable for surgical applications involving parenchymal organ bleeding, but certain products may perform better than others, including Cutanplast gelatin powder in diffuse mild bleeding (such as liver abrasion), and Cutanplast Fast gelatin sponge and Emosist ORC gauze for splenic bleeding.

## Background

Bleeding is a frequent complication of surgery that can lead to substantial morbidity and mortality, longer surgeries and hospital stays, and increased total hospital costs [1–5]. Use of meticulous intraoperative hemostasis to reduce the risk of bleeding complications can be expected to have tremendous clinical and economic benefits [1, 3]. In addition to conventional surgical methods (i.e., suturing and electrocautery), absorbable topical hemostatic agents can be used to improve surgical hemostasis [1, 6].

A variety of absorbable topical hemostats, including mechanical hemostats, are available to help control bleeding during a range of surgical procedures [7–11]. When directly applied to the bleeding surface, mechanical hemostats, which are appropriate for use in patients with an intact coagulation system, provide a barrier to stop the flow of blood and a surface that passively promotes platelet activation and aggregation, and assists clot formation [8–11]. Mechanical hemostats are among the easiest to use, safest, and most low-cost hemostats available [12–14].

Originally introduced over 60 years ago, gelatin- and oxidized regenerated cellulose (ORC)-based devices are two of the most commonly used mechanical hemostats [7, 8, 15]. Gelatin hemostatic devices are manufactured from animal-derived gelatin, and are available in sponge, powder, and granular forms that are highly absorptive and largely non-antigenic [7, 8]. Gelatin sponge products differ with respect to gelatin density and porosity, which affects their biochemical and biological effects [16–18]. ORC hemostatic products contain plant-derived continuous cellulose fibers in fabric formats that are easier to handle than gelatin sponge products, and as opposed to pH-neutral gelatin products, have a pH-lowering effect that contributes to hemostatic and bactericidal properties [7, 8, 10]. Thanks to these features, ORC products are especially well known and widely used [19–21].

Developed in 1998, Cutanplast (Mascia Brunelli S.p.A., Milan, Italy) is a novel highly absorbent and porous gelatin product with a powerful hemostatic effect [16–18, 22–24]. Rapid hemostasis may be explained by platelet adhesion and activation of the natural coagulation system on the porous surface of the gelatin sponge [22, 23]. In addition to the original Cutanplast products (Cutanplast Standard), newer, relatively high porosity Cutanplast Fast sponge and powder products, with improved absorbency and hemostatic capacity, due to their higher porosity structure, as well as handling characteristics, are now available. The aim of this study was to evaluate the hemostatic efficacy of Cutanplast Standard and Fast gelatin sponge and powder products and Emosist ORC gauze in porcine liver and spleen models of surgical bleeding.

## Methods

### Hemostatic devices

All five hemostat devices used in this study (Cutanplast Standard and Fast gelatin sponge products, Cutanplast Standard and Fast gelatin powder products and Emosist ORC gauze) are manufactured by Mascia Brunelli S.p.A., Milan, Italy. All products are supplied in sterile packaging. Cutanplast Standard gelatin sponge is prepared by presoaking in sterile saline solution and squeezing slightly before use. Cutanplast Standard and Fast gelatin powder products are prepared by adding 6 mL of sterile saline solution and until a soft and moldable paste is obtained. Cutanplast Fast gelatin sponge and Emosist ORC gauze are ready to use dry.

### Animal experiments

The experimental procedures performed in this study were conducted in accordance with Italian law and European Union Directive 2010/63/UE on the protection of animals used for scientific purposes. The study received ethical approval from the Italian Ministry of Health (Authorization number 367–2015-PR). The animals were obtained with permission from the Biotechnology Research Center for Cardiothoracic Applications (CRABCC, Rivolta d’Adda, Cremona, Italy).

Five female Landrace-Large White pigs (average weight 75–80 kg) were used. Porcine models of liver abrasion, incision, and punch biopsy have previously been used to assess the efficacy of gelatin and ORC hemostat products for diffuse and focused mild-to-moderate surgical bleeding [19–21, 25, 26]. Before the surgical procedures, the animals had an acclimation and observation period of 10 days, during which they were housed under standard conditions (a light-dark cycle of 12:12 h, temperature of 20 °C, and humidity of 50 ± 5%), with freely available food and water. Before the surgical procedures, animals were fasted for 10 h.

Each pig received an intramuscular injection of ketamine 10 mg/kg and midazolam 0.5 mg/kg, followed by inhalation anesthesia with a mixture of isoflurane 4.5% and oxygen. When a sufficient level of anesthesia was achieved, animals were intubated and anesthesia was maintained with isoflurane (2.5%) mixed with 100% oxygen. When necessary, curarization was obtained with an atracurium bolus (2 mg/kg) and maintained with a 0.12 mg/kg/min infusion. Animals were mechanically ventilated with a tidal volume of 10 mL/kg, a positive end-expiratory pressure of 5 cmH_2_0 and a respiratory rate required for normocapnia (pCO_2_ 35–45 mmHg). Intraoperative analgesia was provided with tramadol (intravenous [IV] 1 mL/kg) and meloxicam (0.4 mg/kg). A catheter was placed in the auricular vein, and IV Ringer’s Solution was administered during the surgery. Vital signs and oxygenation were continuously monitored (electrocardiography, SpO_2_ and invasive blood pressure using a transducer connected to a catheter placed in the auricular artery). Upon completion of the procedures, animals were humanely euthanized with an IV overdose of potassium chloride under deep anesthesia.

### Surgical procedures

All surgical procedures were performed under the same sterile conditions. Animals were placed in the ventrodorsal recumbant position and a mini-laparotomy was performed. The skin incision began below the xiphoid process and extended 15 cm in the cranioventral direction. The linea alba was opened, the liver was exposed, and the Balfour self-retaining abdominal retractor was used to maintain access to the liver and spleen. Throughout each procedure, the liver and spleen were kept moist with saline-soaked laparotomy sponges.

**Fig. 1:**
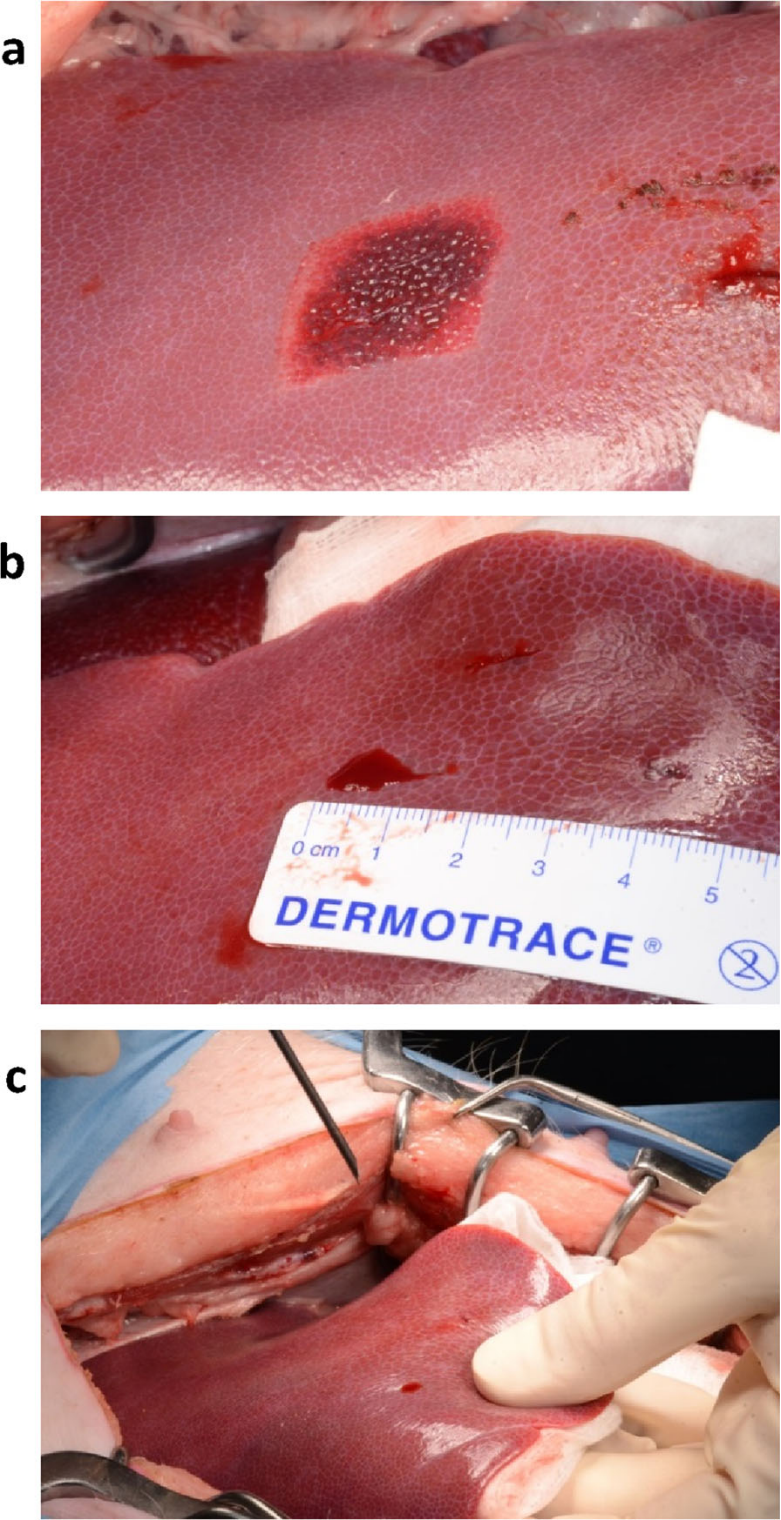
Images of the surgical injuries applied to the liver, including (**a**) abrasion, (**b**) incision, and (**c**) puncture injuries

There were five types of surgical injury: liver abrasion, incision and puncture (Fig. 1), and spleen incision and puncture. In the liver abrasion model, a scalpel blade was used to abrade the liver surface to create 2.0 × 2.0 cm lesions, with application of the right amount of pressure needed to break through the visceral peritoneum and fibrous liver capsule to expose the liver parenchyma and cause sustained, uniform bleeding (mild hemorrhage or oozing). Incisions measuring 1.0 cm in length and 6–7 mm deep were made with a scalpel. Puncture wounds (6–7 mm deep) were made with a 12-gauge needle. All five hemostat devices (Cutanplast Standard and Fast gelatin sponge products, Cutanplast Standard and Fast gelatin powder products and Emosist ORC gauze) were tested in the liver abrasion and incision injuries, but only the Cutanplast Standard and Fast sponge products and Emosist ORC gauze were tested in the liver puncture and spleen incision and puncture injuries. For each animal, there was one injury for each product tested in each surgical model. Therefore, in total, there were 13 liver injury sites (five abrasion, five incision and three puncture sites) and six spleen injury sites (three puncture and three incision sites) per animal.

**Fig. 2:**
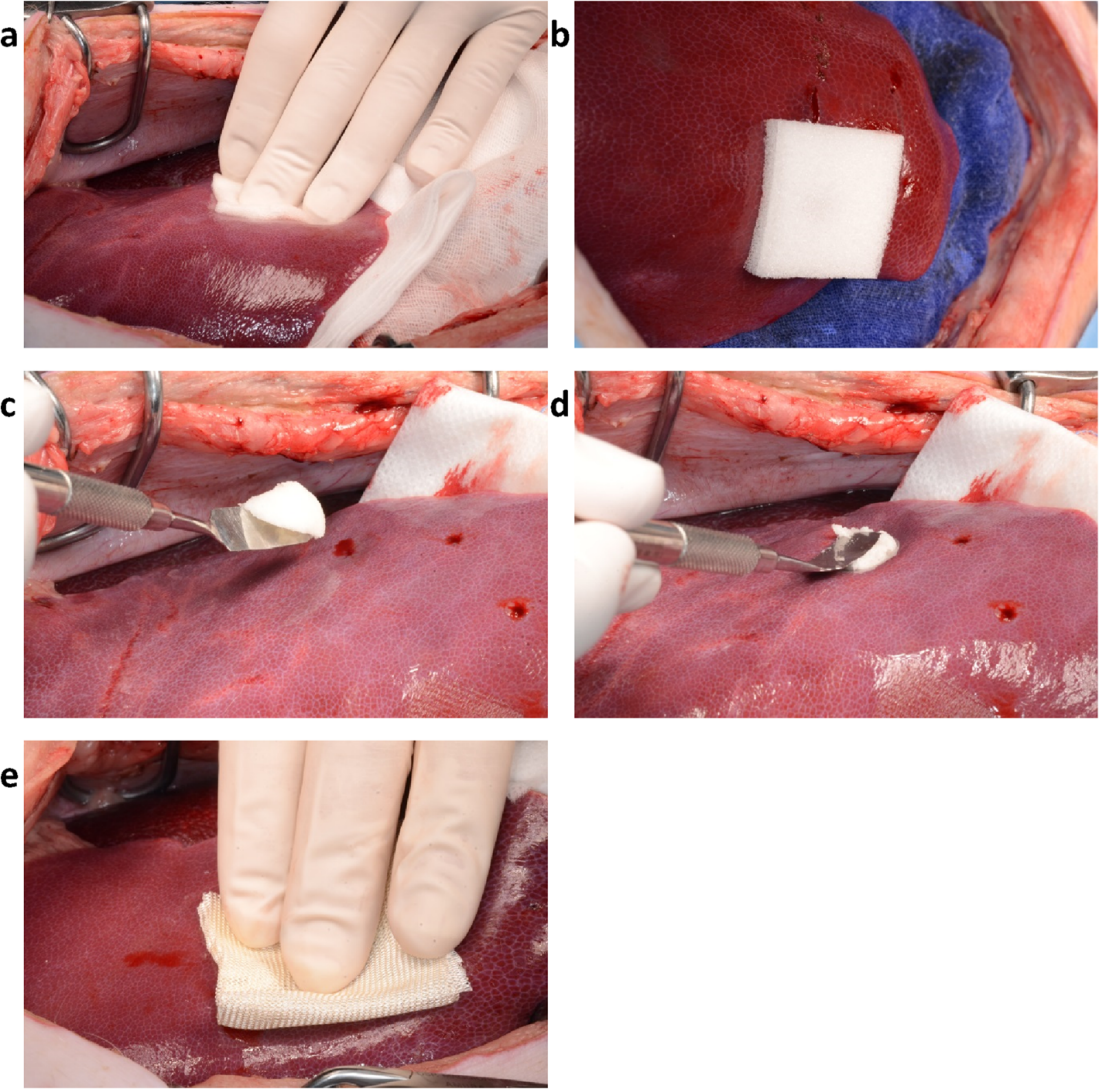
Application of the hemostat products: (**a**) Cutanplast Standard sponge, (**b**) Cutanplast Fast gelatin sponge, (**c**) Cutanplast Standard powder, (**d**) Cutanplast Fast gelatin powder, and (**e**) Emosist oxidized regenerated cellulose gauze

Products were prepared and applied according to prescribing information/instructions for use. Injuries were left to bleed for 10 s before applying the hemostat products. Cutanplast gelatin sponge products and Emosist ORC gauze were cut into 3.5 × 3.5 cm squares, with the ORC gauze folded into a double layer before being trimmed to size. After presoaking in sterile saline solution and squeezing, Cutanplast Standard sponge was applied using slight pressure (Fig. 2a). Cutanplast Fast gelatin sponge was applied dry without pressure (Fig. 2b). After preparing the Cutanplast Standard and Fast gelatin powder products (addition of 6 mL of sterile saline solution to the packaging bottle and mixing for 30 s), the resulting paste was applied to the surgical wound using a spatula without any pressure (Fig. 2c and d). Emosist ORC gauze was applied dry without any pressure (Fig. 2e). For removal, gelatin sponge and ORC products could be peeled away from the injured area, and paste (powder) products could be washed away from the injury using a syringe of sterile water.

### Assessment of bleeding and quantification of blood loss

Bleeding was evaluated using an adapted version of a validated intraoperative bleeding severity scale, which classifies bleeding into grades according to visual presentation, anatomic appearance, qualitative description and visually estimated rate of blood loss (Table 1) [27]. An additional parameter (time to hemostasis) was included in the scale. Qualitative parameters (visual presentation, anatomic appearance, and qualitative description) were evaluated 2 min after application of the hemostatic device. These parameters were graded on a 5-point Likert-type scale, ranging from 0 (spurting or gushing, central arterial- or venous-like bleeding of a life-threatening nature) to 4 (no bleeding) by the same three assessors for each pig. Hemostasis was defined as the absence of observable active bleeding or the absence of sustained soaking of bleed into the hemostatic material. If hemostasis was not achieved 2 min after application of the hemostatic device, the rate of blood loss was quantified using dry pre-weighted gauze to collect blood for 1 min. The hemostatic products were applied for the time necessary to stop bleeding for up to 10 min. This cut-off point for the evaluation of bleeding time was chosen based on examples of previous models of hepatic bleeding [25, 26, 28]. If required, new hemostats were applied thereafter.

**Table 1:** Adapted version of the validated intraoperative bleeding scale. Adapted with permission from Lewis KM, et al. Surgery. 2017 [27]

Grade	Visual presentation	Anatomic Appearance	Qualitative description	Time to Hemostasis^**a**^ (min)	Rate of blood loss^**b**^ (mL/min)
**4**	No bleeding	No bleeding	No bleeding	≤ 2	≤ 1
**3**	Ooze or intermittent flow	Capillary-like bleeding	Mild	> 2–5	> 1–5
**2**	Continuous flow	Venule and arteriolar-like bleeding	Moderate	> 5–8	> 5–10
**1**	Controllable spurting and/or overwhelming flow	Non central venous and arterial-like bleeding	Severe	> 8–10	> 10–50
**0**	Unidentified or inaccessible spurting or gush	Central arterial- or venous-like bleeding	Life-threatening^c^	> 10	> 50

^a^Parameter added to the original four items of the intraoperative bleeding scale, which was designed and validated for use in clinical studies to generate labelling claims [27]

^b^Visual rate of blood loss (original item of the intraoperative bleeding scale) [27], unless hemostasis was not achieved 2 min after application of the hemostatic device, in which case the rate of blood loss was quantified using dry pre-weighted gauze to collect blood loss for 1 min

^c^Systemic resuscitation required (e.g., volume expanders, vasopressors, or blood products)

### Statistical analysis

For each bleeding rating scale item, the grades obtained from the three assessors were averaged for each animal, and contributed to the final results, which were expressed as mean values ± standard deviation.

## Results

Hemostasis was achieved within 10 min of application of all hemostats for all injuries in all models of bleeding.

### Liver abrasion and incision

**Fig. 3:**
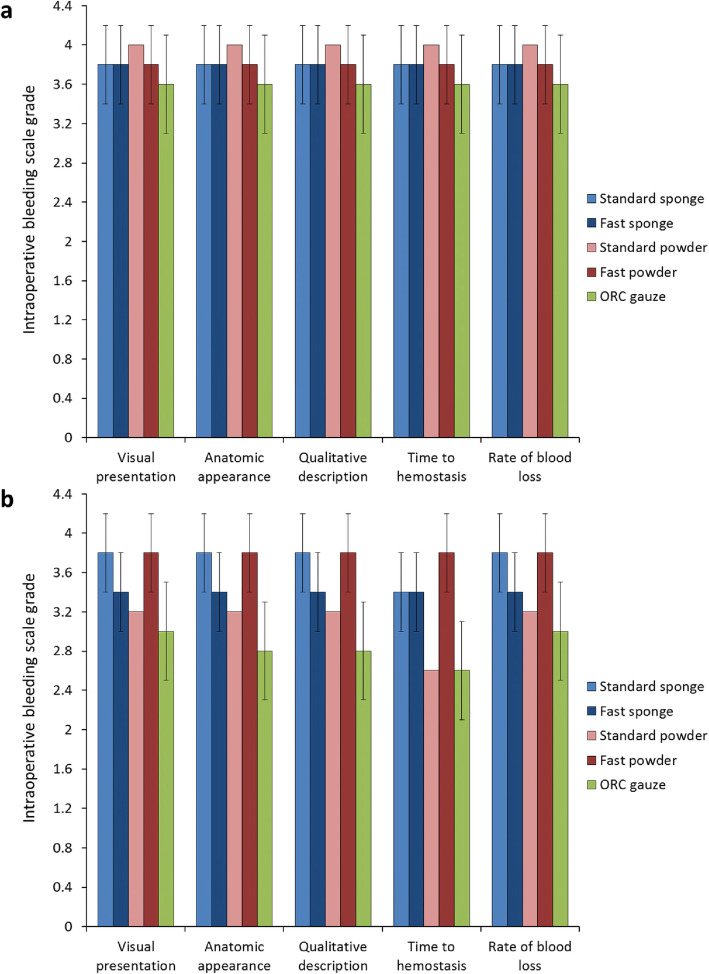
Intraoperative bleeding scale grades (mean ± standard deviation) for (**a**) liver abrasion and (**b**) liver incision treated with Cutanplast Standard or Fast gelatin sponge, Cutanplast Standard, or Fast gelatin powder or Emosist oxidized regenerated cellulose gauze

All five intraoperative bleeding scale parameters (visual presentation, anatomic appearance, qualitative description, time to hemostasis, and rate of blood loss) were graded as 4.0 after application of Cutanplast Standard gelatin powder in the liver abrasion model (Fig. 3). Ratings for the other gelatin-based products were consistently graded 3.8, and Emosist ORC gauze was consistently graded 3.6.

With the exception of time to hemostasis with Cutanplast Standard sponge (grade 3.4), grade 3.8 ratings were consistently obtained with Cutanplast Standard sponge and Fast powder in the liver incision model (Fig. 3). Time to hemostasis was graded 3.4 for Cutanplast Fast sponge, and 2.6 for Cutanplast Standard powder and Emosist ORC gauze. For all other parameters, rating grades were 3.2–3.4 for the Cutanplast Fast sponge and Standard powder gelatin-based products, and 2.8–3.0 for Emosist ORC gauze.

### Liver puncture

**Fig. 4:**
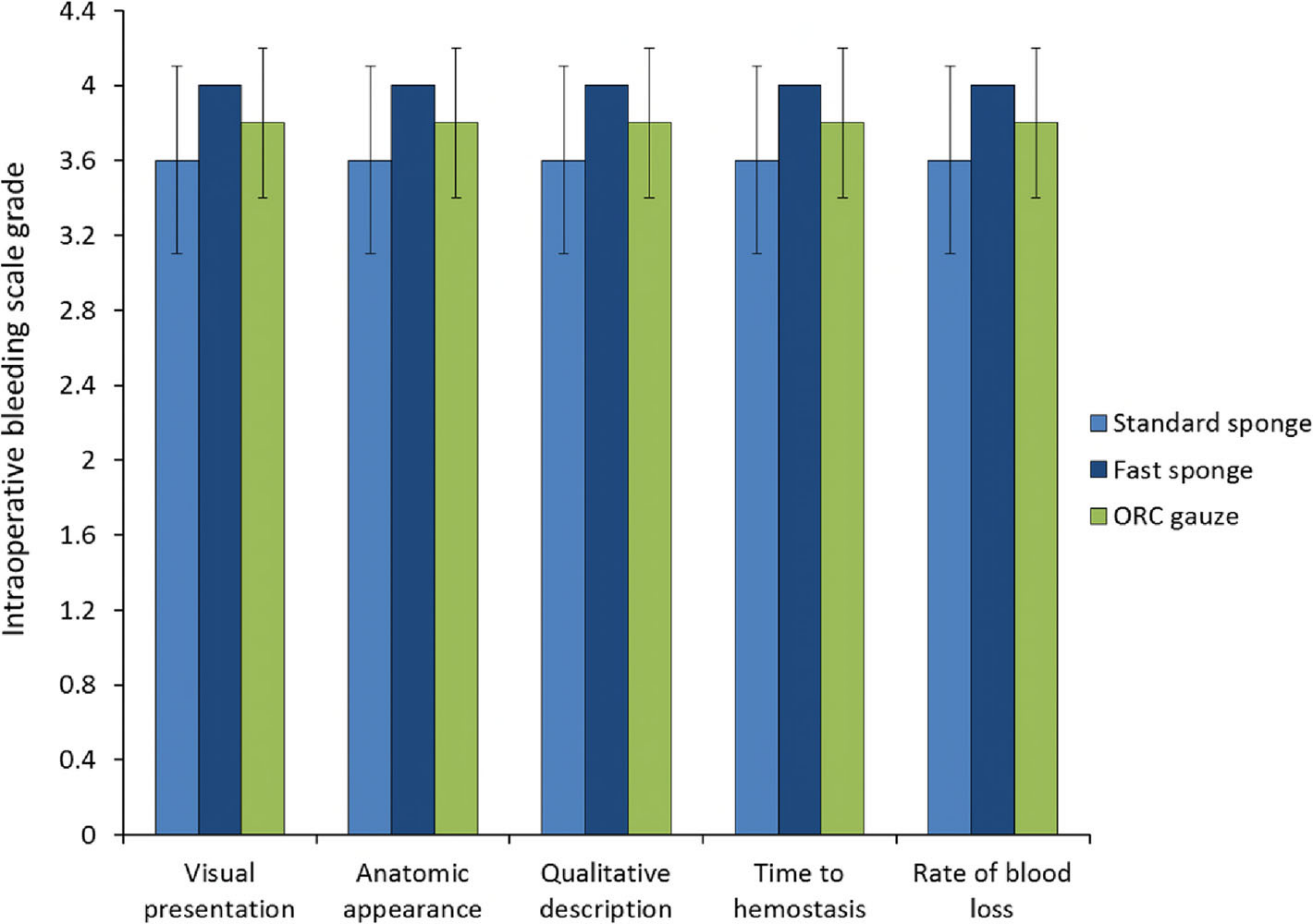
Intraoperative bleeding scale grades (mean ± standard deviation) for liver puncture treated with Cutanplast Standard or Fast gelatin sponge or Emosist oxidized regenerated cellulose gauze

Intraoperative bleeding scale ratings of 4.0 were achieved with Cutanplast Fast gelatin sponge for all parameters in the liver puncture model (Fig. 4). Ratings were consistently grade 3.6 and 3.8 with Cutanplast standard sponge and Emosist ORC gauze, respectively.

### Spleen incision and puncture

**Fig. 5:**
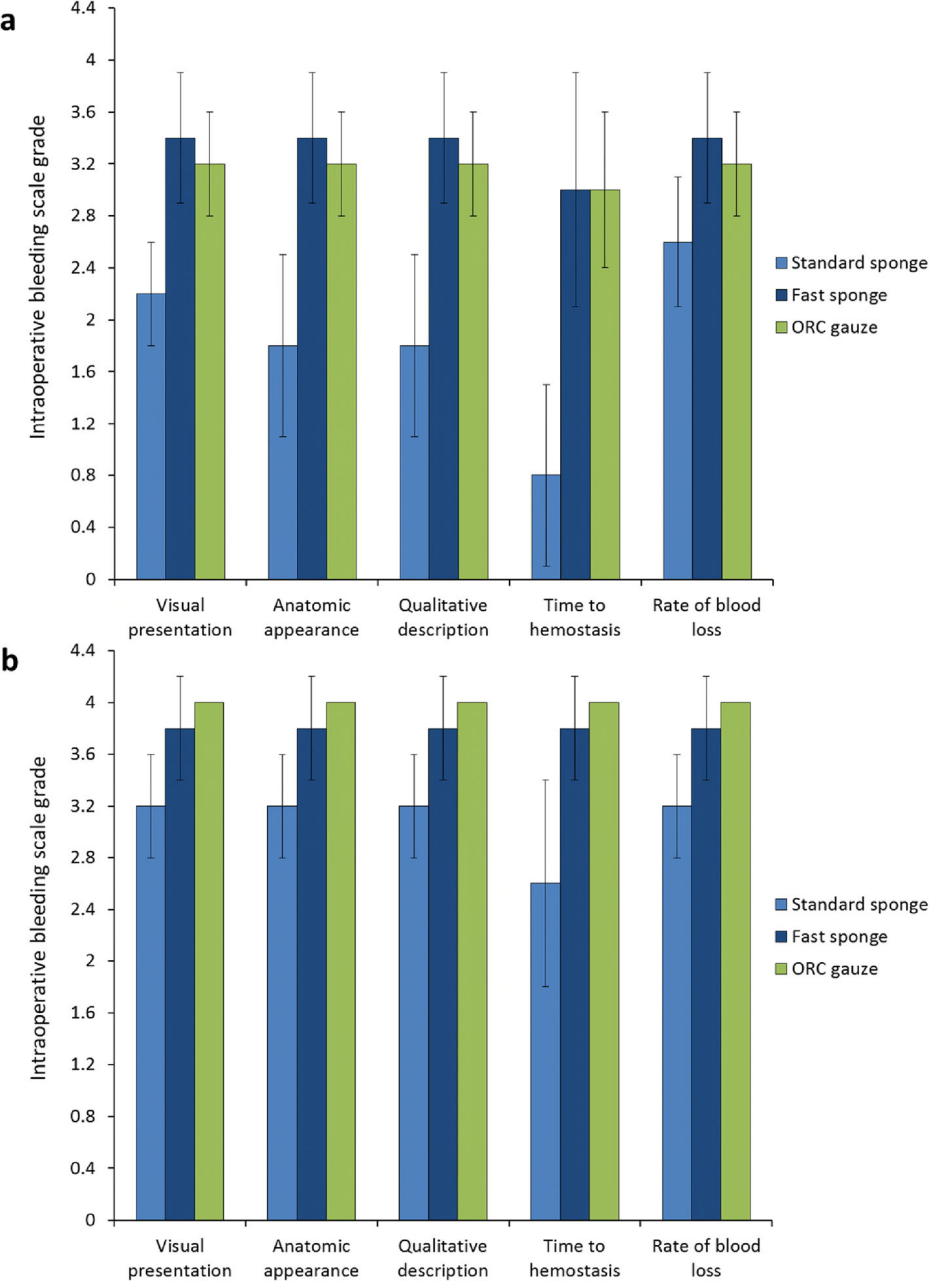
Intraoperative bleeding scale grades (mean ± standard deviation) for (**a**) spleen incision and (**b**) spleen puncture treated with Cutanplast Standard or Fast gelatin sponge or Emosist oxidized regenerated cellulose gauze

Time to hemostasis was graded 3.0 for both the Cutanplast Fast gelatin sponge product and Emosist ORC gauze in the spleen incision model, and other intraoperative bleeding rating scale items were consistently graded 3.2 to 3.4 for these products (Fig. 5). Grade 0.8 time to hemostasis was obtained with the Cutanplast Standard gelatin sponge product in this model, and other intraoperative bleeding rating scale items were graded 1.8 (anatomic appearance and qualitative description) to 2.6 (rate of blood loss) for this product.

Grade 4.0 ratings were consistently achieved for all intraoperative bleeding rating scale parameters with Emosist ORC gauze in the spleen puncture model (Fig. 5). Grade 3.8 ratings were consistently obtained for the Cutanplast Fast gelatin sponge product, and with the exception of time to hemostasis (grade 2.6), bleeding rating scale items were all grade 3.2 after application of Cutanplast Standard gelatin sponge.

### Comparison of time to hemostasis

**Fig. 6:**
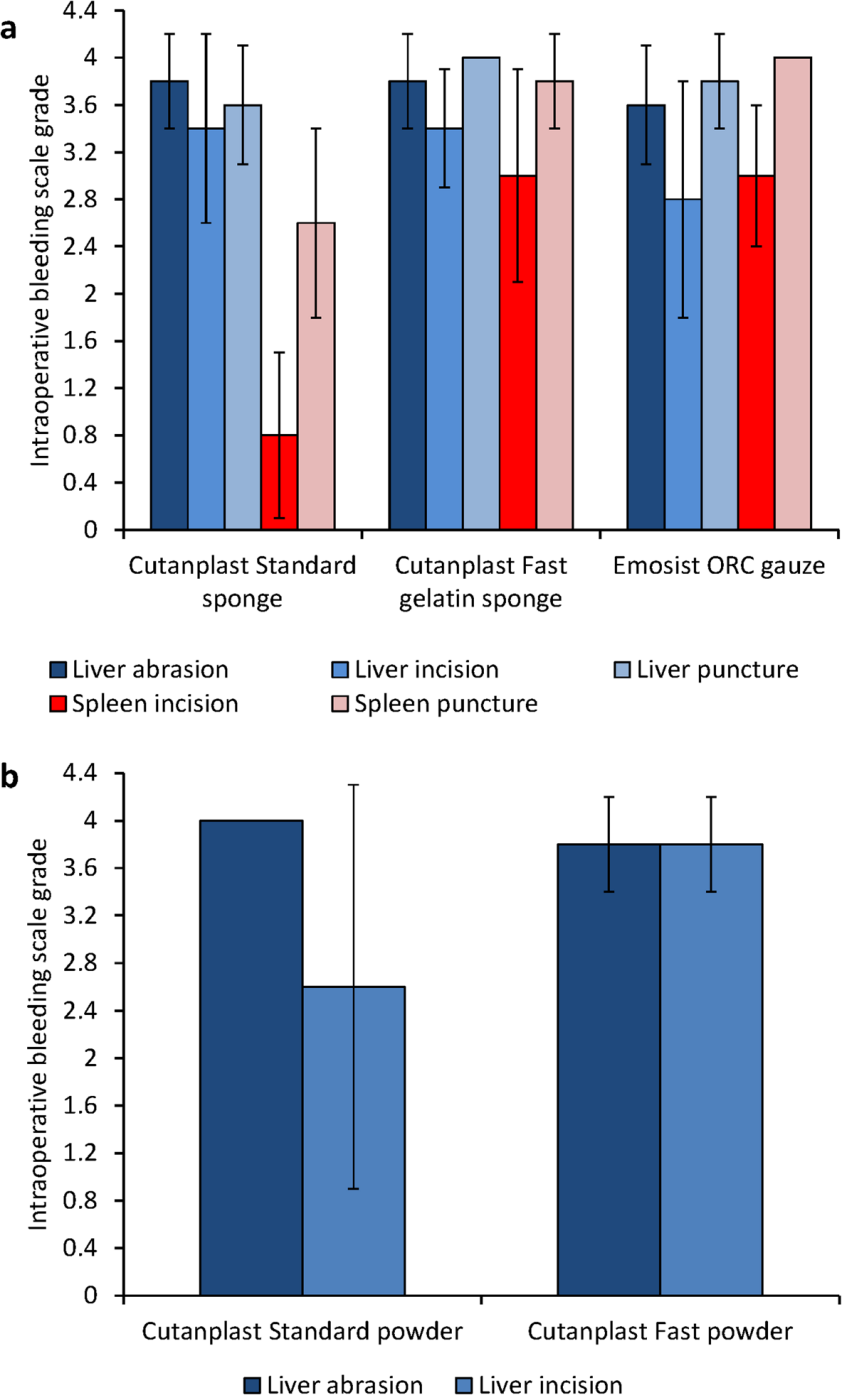
Comparison of time to hemostasis intraoperative bleeding scale grades (mean ± standard deviation) for (**a**) Cutanplast Standard and Fast gelatin sponges, and Emosist oxidized regenerated cellulose gauze in liver abrasion, incision, and puncture and spleen incision and puncture models, and (**b**) Cutanplast Standard and Fast powder in liver abrasion and incision models

When comparing time to hemostasis intraoperative bleeding scale grades for Cutanplast Standard sponge, Cutanplast Fast gelatin sponge, and Emosist ORC gauze for each type of surgical injury, the efficacy for treating small bleeding sites (e.g. liver abrasion) for all products seemed to be similar (grade 3.6–3.8; Fig. 6a). Across the different surgical injury types, Cutanplast Standard sponge had greater efficacy for liver than spleen bleeding models (grade 3.4–3.8 vs grade 0.8–2.6), Emosist ORC gauze showed the greatest efficacy for surgical puncture liver and spleen models (grades 3.8 and 4.0, respectively), and Cutanplast Fast gelatin sponge showed efficacy across all models of surgical injury (grade 3.0–4.0).

Both the Cutanplast Standard and Cutanplast Fast powder products showed similar efficacy for the liver abrasion model (intraoperative bleeding scale grade 3.8–4.0), but Cutanplast Fast powder had better efficacy than Cutanplast Standard powder for the treatment of liver incision (grade 3.8 vs 2.6; Fig. 6b).

## Discussion

We investigated the hemostatic efficacy of Cutanplast Standard and Fast gelatin sponge products and Emosist ORC gauze in a range of porcine models of mild-to-moderate surgical bleeding that are expected to be predictive of clinical efficacy. These were porcine liver abrasion, incision, and puncture, and spleen incision and puncture models. Cutanplast gelatin powder products were only evaluated in porcine liver abrasion and incision models. We do not consider our liver puncture or spleen puncture and incision models relevant to the clinical conditions for which powdered topical hemostats are indicated (mild bleeding or oozing from a relatively diffuse bleeding surface).

In our liver abrasion and incision models, rapid hemostasis (generally ≤2–5 min) was achieved with all Cutanplast gelatin sponge and powder products, which were at least as effective as Emosist ORC gauze. Cutanplast Standard gelatin powder was particularly effective in the liver abrasion model, with no observed bleeding and time to hemostasis ≤2 min. Hemorrhage was also well controlled with Cutanplast Standard and Fast gelatin sponge products and Cutanplast Fast gelatin powder in the liver incision model, with mild-to-no capillary-like bleeding with ooze or intermittent flow, and time to hemostasis of ≤2–5 min, whereas time to hemostasis tended to be > 5 min with Cutanplast Standard gelatin powder and Emosist ORC gauze in this model. Inferior hemostasis occurred with Cutanplast Standard gelatin sponge versus Cutanplast Fast gelatin sponge and Emosist ORC gauze in the liver puncture and spleen puncture and incision models. This was most evident for the spleen incision model, in which a continuous flow of blood was observed and time to hemostasis approached 10 min with Cutanplast Standard gelatin sponge, whereas ooze or intermittent flow was observed with Cutanplast Fast gelatin sponge and Emosist ORC gauze products, with hemostasis taking place in 2–5 min.

Surgical bleeding can range from mild or moderate in intensity to severe or traumatic [1]. In general, topical hemostats can be used to achieve hemostasis on the surface of parenchymal organs, but other methods of hemostasis are recommended when there is a relatively large amount of bleeding (i.e., pulsatile/spurting arterial or high volume venous bleeding rather than mild-to-moderate hemorrhage or diffuse oozing) [9, 20]. Porcine liver abrasion, incision, and punch biopsy models have previously been used to assess the efficacy of gelatin and ORC hemostat products on diffuse and focused mild-to-moderate surgical bleeding [19–21, 25, 26]. The effectiveness of gelatin and ORC hemostatic products has also previously been demonstrated in relation to injuries to the spleen, which is an organ where hemostasis is relatively difficult to achieve [29–31]. As opposed to abrasions and shallow incisions (2–3 mm), which generally result in a mixture of venous-, venule-, and arteriolar-like bleeding [19], our 6–7-mm deep liver and spleen incision models mimicked a relatively severe surgical injury, with more potential for flowing venous and/or arterial bleeding.

During surgery, effective hemostasis through the adjunctive use of hemostatic agents can reduce operating times, blood loss, and the need for transfusions [32, 33]. However, the comparative effectiveness of topical hemostatic agents is highly procedure specific [6], and the most appropriate product needs to be selected, taking into consideration factors such as the intensity of bleeding, surgical site, and type of surgical procedure [2, 5, 10, 13, 32, 34]. To maximize effectiveness and reduce the risk of hemostatic agent failure, comparison of efficacy of hemostatic agents used in routine surgery is important in order to guide selection of the appropriate hemostatic agent [21, 27]. To date, Cutanplast gelatin sponge has been shown to be a clinically effective hemostat in endoscopic sinus surgery and thyroid surgery [16–18], and Emosist ORC gauze ensured adequate hemostasis in patients undergoing laparoscopic cholecystectomy with bleeding not adequately controlled by conventional techniques [9]. Our in vivo observations suggest that these products could also be used successfully in other surgical applications.

The current study was purely observational with a small sample size (five pigs). It was designed to support in vitro studies on the efficacy of our hemostatic products using in vivo models that are relevant to clinical practice. There were therefore no formal statistical comparisons of the hemostatic effectiveness of the different hemostatic products. In addition to the qualitative parameters of blood loss, time to hemostasis was assessed (an important factor to consider when choosing a topical hemostat [30]), and the rate of blood loss was quantified if time to hemostasis was not achieved within 2 min of application of the hemostatic device. These two latter quantitative parameters were designed to support subjective and potentially inaccurate visual estimation of blood loss.

## Conclusions

Taking into account their inherent limitations, our in vivo observations suggest that Cutanplast Standard and Fast gelatin sponge and powder products may be well suited to general surgical situations mimicked by our liver incision and abrasion models (i.e., partial liver resection with mild-to-moderate hemorrhage or ooze from the resected surface). The powder form may be particularly useful in diffuse mild bleeding scenarios or when it would be difficult to position a gel or fabric in place. Cutanplast Fast gelatin sponge and Emosist ORC gauze, which demonstrated relatively fast and more effective hemostasis in spleen incision and puncture models, may be more useful than Cutanplast Standard gelatin sponge as adjunctive agents in similar clinical practice conditions (i.e., spleen trauma/injury).

## Data Availability

The datasets generated during and/or analyzed during the current study are available from the corresponding author on reasonable request.

## Abbreviations

IV: Intravenous
ORC: Oxidized Regenerated Cellulose
